# Navigating Haploidentical Stem Cell Transplantation in Congenital Amegakaryocytic Thrombocytopenia With Prior Intracranial Bleeding: Report of Two Pediatric Cases

**DOI:** 10.7759/cureus.106037

**Published:** 2026-03-28

**Authors:** Aditi Tulsiyan, Nita Radhakrishnan, Anuj Singh, Sudipto Bhattacharya, Hari Gaire, Satyam Arora, Seema Dua, Savitri Singh, Anukriti Srivastava

**Affiliations:** 1 Department of Pediatric Hematology Oncology, Post Graduate Institute of Child Health, Noida, IND; 2 Department of Transfusion Medicine, Post Graduate Institute of Child Health, Noida, IND; 3 Department of Pathology, Post Graduate Institute of Child Health, Noida, IND

**Keywords:** congenital amegakaryocytic thrombocytopenia, haploidentical hsct, haploidentical stem cell transplantation, intracranial hemorrhage, mpl mutation

## Abstract

Congenital amegakaryocytic thrombocytopenia (CAMT) is a rare inherited bone marrow failure syndrome caused by biallelic myeloproliferative leukemia (MPL) variants, for which hematopoietic stem cell transplantation (HSCT) is the only curative therapy. We report two children with genetically confirmed CAMT and prior intracranial hemorrhage who underwent haploidentical peripheral blood HSCT with reduced intensity conditioning and post-transplant cyclophosphamide. Individualized management included immunomodulation (bortezomib for alloimmune platelet refractoriness in one child; rituximab for B-cell depletion in the other) and an intensified platelet transfusion strategy to maintain hemostatic stability during the peri-transplant period. Both achieved prompt engraftment, transfusion independence, and sustained donor chimerism without neurological deterioration. These cases demonstrate the feasibility of haploidentical HSCT in high-risk CAMT.

## Introduction

Congenital amegakaryocytic thrombocytopenia (CAMT; OMIM 604498) is an exceptionally rare inherited bone marrow failure syndrome characterized by severe thrombocytopenia from birth, progressive pancytopenia, and a marked reduction or absence of megakaryocytes in the bone marrow. The disorder results from biallelic pathogenic variants in the myeloproliferative leukemia (MPL) gene, which encodes the thrombopoietin receptor (c-Mpl), leading to defective megakaryopoiesis and eventual depletion of hematopoietic stem cells [1-3].

CAMT typically presents in early infancy with petechiae, purpura, or life-threatening bleeding. Most untreated children develop bone marrow failure within the first few years of life, although the rate of progression depends on the underlying MPL genotype: Type I (null mutations) leads to early pancytopenia, while Type II (hypomorphic mutations) may allow transient hematopoietic recovery before eventual aplasia [4-6]. 

The global incidence of CAMT is unknown, with fewer than 100-200 well-characterized cases reported worldwide. Data from the German Registry for Inherited Bone Marrow Failure Syndromes indicate that CAMT accounts for less than 1% of pediatric bone marrow failure cases [7]. Published experience on CAMT remains limited to isolated case reports and small institutional series [8,9]. Without hematopoietic stem cell transplantation (HSCT), prognosis remains poor, as most affected children succumb to bleeding or marrow failure in early childhood. HSCT remains the only curative option. Early experience with matched sibling or unrelated donor HSCT demonstrated variable outcomes, with transplant-related mortality approaching 30-40% in historical series [10-14]. However, advances in understanding of conditioning intensity, graft manipulation, and post-transplant immunosuppression have led to markedly better survival in recent years [11,12].

Haploidentical HSCT using T cell-replete grafts with post-transplant cyclophosphamide (PTCy) has revolutionized donor accessibility, offering a curative approach for children lacking HLA-matched donors. Nevertheless, published reports of haplo-HSCT in CAMT are sparse, and data regarding transplantation in children with severe bleeding phenotypes, such as prior intracranial hemorrhage (ICH), are virtually nonexistent [14-17]. ICH is a devastating complication of CAMT, reported in up to 15-20% of affected infants. These catastrophic events often result in early mortality or long-term neurological sequelae and may delay definitive curative therapy [6].

In this report, we describe two children with genetically confirmed CAMT who had experienced intracranial bleeding prior to transplantation and subsequently underwent successful haploidentical peripheral blood HSCT. We discuss the unique clinical challenges associated with transplantation in this high-risk subset and emphasize that timely genetic diagnosis, early donor identification, and meticulous peri-transplant management can enable life-saving outcomes even in children with pre-existing life-threatening bleeding and transfusion dependence.

## Case presentation

This report describes the experience of haploidentical hematopoietic stem cell transplantation (HSCT) in two pediatric patients with genetically confirmed congenital amegakaryocytic thrombocytopenia (CAMT), both with a history of intracranial bleeding. Data were collected prospectively from clinical, laboratory, and transplant records during HSCT. Written informed consent was obtained from the parents for the procedure and for the publication of anonymized data. Institutional ethics approval was waived as this report includes only de-identified clinical data from routine care; written informed consent for publication was obtained from the patients’ guardians, in accordance with the Declaration of Helsinki. The details of both cases are represented in Table 1.

**Table 1:** Salient clinical and transplant features of both patients with congenital amegakaryocytic thrombocytopenia Abbreviations: ANC = absolute neutrophil count; CAMT = congenital amegakaryocytic thrombocytopenia; MPL: myeloproliferative leukemia; CMV = cytomegalovirus; GVHD = graft-versus-host disease; HSCT = hematopoietic stem cell transplantation; ICH = intracranial hemorrhage; MMF = mycophenolate mofetil; PTCy = post-transplant cyclophosphamide; rATG = rabbit anti-thymocyte globulin; TBI = total body irradiation

Parameter	P1	P2
Age at HSCT	3.5 years	5 years
Sex	Male	Male
Parental consanguinity	No	Yes
Family history	Elder sibling died with aplastic anemia (likely CAMT)	None reported
Initial presentation	Thrombocytopenia	Thrombocytopenia
Age at presentation	2 months	3 months
Bone marrow findings	Absent megakaryocytes; evolved to aplastic anemia	Absent megakaryocytes
Genetic variant (MPL)	c.235_236del (p.Leu79GlufsTer84); homozygous	c.212+5G>A (splice-site); homozygous
Molecular classification	Pathogenic (type I CAMT)	Pathogenic (type I CAMT)
Phase at HSCT	Complete marrow aplasia, platelet refractoriness	Platelet transfusion dependent
Prior intracranial bleeding	Spontaneous intracranial hemorrhage at 2 years	Intracranial hemorrhage with sequelae at 5 years
Serum ferritin prior to HSCT	4290 ng/mL	1600 ng/mL
Donor	Father (5/10 HLA match)	Father (5/10 HLA match)
Blood group (donor/recipient)	B positive / B positive	B positive / B positive
CMV serostatus (donor/recipient)	Negative / negative	Positive / positive
Conditioning regimen	Fludarabine, cyclophosphamide, rATG, TBI (2 Gy)	Rituximab, fludarabine, cyclophosphamide, rATG, TBI (4 Gy)
Stem cell source	Peripheral blood stem cells	Peripheral blood stem cells
CD34 cell dose	8.2×10⁶/kg	18×10⁶/kg
CD3 dose	3x10^8^/kg	2x10^8^/kg
GVHD prophylaxis	PTCy, cyclosporine, MMF	PTCy, cyclosporine, MMF
Neutrophil engraftment (ANC > 0.5 × 10⁹/L)	Day +14	Day +14
Platelet independence	Day +14	Day +11
Acute GVHD	Grade II skin, steroid-responsive	Grade II gut, steroid-responsive
Veno-occlusive disease	None	None
CMV reactivation during HSCT	Yes	No
Duration of hospital stay	32 days	34 days
Status at discharge	Engrafted, transfusion independent, neurologically intact	Engrafted, transfusion independent, neurologically intact
Status at last follow-up on 15 March 2026	Alive, full donor chimerism Day +555	Alive, full donor chimerism Day +238

Case 1

The first patient was a 3.5-year-old boy, the second child of non-consanguineous parents, who presented at two months of age with petechiae and severe thrombocytopenia. His elder sibling had been diagnosed at our center three years earlier with aplastic anemia at five years of age. As chromosomal breakage studies for Fanconi anemia were negative, she received immunosuppressive therapy with cyclosporine and anti-thymocyte globulin but failed to respond and died of pulmonary hemorrhage. Whole-exome sequencing was not performed for her, and she may have represented an undiagnosed case of CAMT.

**Figure 1:**
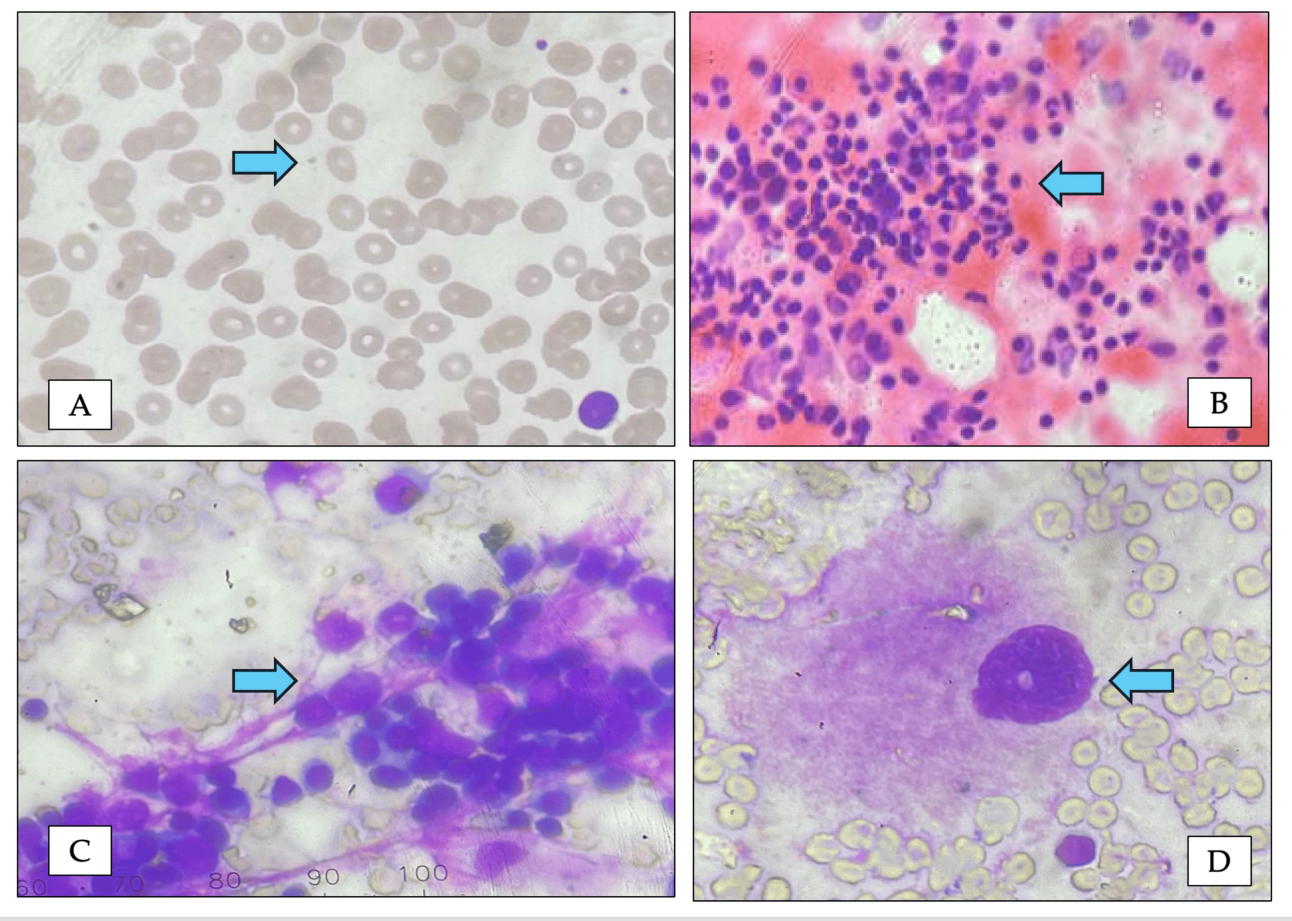
Peripheral smear and bone marrow findings of P1 (A) Peripheral blood smear (Leishman stain, ×40) showing severe thrombocytopenia. (B) Bone marrow aspirate smear (hematoxylin and eosin stain, ×40) demonstrating cellular marrow trails with absence of megakaryocytes. (C) Bone marrow imprint smear (Leishman stain, ×40) showing preserved erythroid and myeloid hematopoiesis with absence of megakaryocytes. (D) Bone marrow aspirate smear (Leishman stain, ×100) demonstrating a dysplastic megakaryocyte with hypolobated nucleus and reduced cytoplasmic granularity, suggestive of impaired thrombopoiesis.

The index patient was noted to have thrombocytopenia at birth, and early evaluation for bone marrow failure syndromes was advised. At 11 months of age, bone marrow examination showed an absence of megakaryocytes with preserved erythroid and myeloid precursors (Figure 1).

Genetic testing identified a homozygous MPL exon 3 frameshift variant (c.235_236del; p.Leu79GlufsTer84), a pathogenic null mutation consistent with CAMT type I, characterized by complete loss of receptor function and early progression to aplasia.

Initially, he was managed with platelet transfusion support. By age three, he had progressed to complete marrow aplasia (<20% cellularity), with transfusion dependence and significant iron overload (serum ferritin 4290 ng/mL). At two years, he experienced a spontaneous intracranial hemorrhage with seizures and altered sensorium. He was managed with daily single-donor apheresis platelet transfusions. In view of poor post-transfusion increments due to alloimmunization, he received four weekly doses of bortezomib, which improved platelet recovery. 

Testing for anti-HLA antibodies was not routinely performed. Platelet refractoriness was clinically suspected based on poor post-transfusion platelet increments after single-donor platelet transfusions, consistent with alloimmune sensitization. In view of poor post-transfusion increments suspected to be due to alloimmunization (poor post-transfusion platelet increment), he received four weekly doses of subcutaneous bortezomib (1.3 mg/m²) as a plasma cell-depleting strategy. This was followed by improvement in post-transfusion platelet increments, allowing safer progression to conditioning. While bortezomib has been used for desensitization in solid organ transplantation, its use in platelet alloimmunization remains off-label and experimental, warranting cautious application.

Baseline cardiac, hepatic, and renal function were normal, and viral serologies (HIV, HBV, CMV) were negative. During pretransplant evaluation, he was found to be hepatitis C virus (HCV) positive, with detectable viral load on quantitative PCR (viral load: 6x10^4^IU/mL). Antiviral therapy with sofosbuvir-velpatasavir was initiated 12 weeks prior to transplantation, and he achieved undetectable HCV RNA by the time of conditioning. Liver function remained stable throughout the peri- and post-transplant period.

In the absence of a matched sibling or unrelated donor, the child underwent haploidentical peripheral blood stem cell HSCT using his father as the donor (5/10 HLA match). Conditioning comprised fludarabine 30 mg/m²/day (days -5 to -1), cyclophosphamide 14.5 mg/kg/day (days -5 and -4), rabbit ATG 1.5 mg/kg/day (days -8 to -6), and total body irradiation (TBI) 200 cGy (day -1). Post-transplant cyclophosphamide (PTCy) 50 mg/kg/day (days +3 and +4) was followed by cyclosporine A (target trough 150-200 ng/mL) and mycophenolate mofetil (MMF) from day +5. The donor was mobilized with G-CSF (10 µg/kg/day for five days), yielding a graft with CD34⁺ cells 8.2×10⁶/kg and a CD3 dose of 3x108/kg. Supportive measures included ursodeoxycholic acid, N-acetylcysteine, and standard antimicrobial prophylaxis.

Engraftment occurred uneventfully, with neutrophil and platelet recovery on day +14. There were no features of veno-occlusive disease. On day +25, he developed fever and skin rash and was diagnosed with grade II acute GVHD, which responded to a short course of prednisolone. Viral PCR surveillance detected CMV viremia (3,859 copies/mL) on day +25, for which ganciclovir was initiated. Therapy was transitioned to valganciclovir after 14 days and continued for 6 weeks, following which it was discontinued after two consecutive negative CMV PCR results. He was discharged on day +32, transfusion-independent and clinically stable. MMF was discontinued on day +28. At last follow-up (day +555; 15 March 2026), he remains well with normal counts, full donor chimerism, and no active GVHD. Cyclosporine is currently being tapered, in line with standard practice for non-malignant transplants.

Case 2

**Figure 2:**
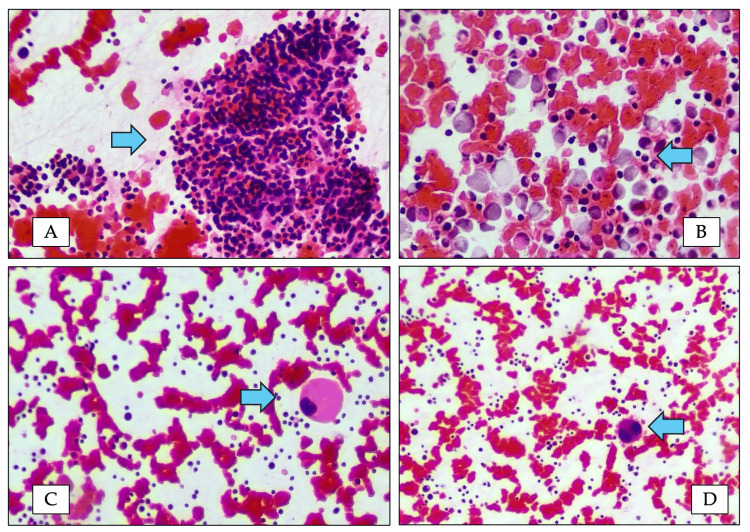
Bone marrow morphology of P2 (A) Bone marrow particle (hematoxylin and eosin stain, ×20) showing age-appropriate cellularity with absence of megakaryocytes. (B) Bone marrow aspirate smear (hematoxylin and eosin stain, ×40) demonstrating cellular marrow trails devoid of megakaryocytes. (C) Bone marrow aspirate smear (Leishman stain, ×40) showing a dysplastic megakaryocyte with nuclear hypolobation. (D) Bone marrow aspirate smear (Leishman stain, ×20) demonstrating a bilobed dysplastic megakaryocyte.

The second patient was a five-year-old boy, born to third-degree consanguineous parents, who presented in early infancy with thrombocytopenia and recurrent mucocutaneous bleeding. Bone marrow aspirate showed reduced megakaryocytes with hypolobation (Figure 2).

Genetic analysis revealed a homozygous MPL intron 2 splice-site variant (c.212+5G>A), classified as likely pathogenic and predicted to disrupt canonical splicing, resulting in loss of receptor function. He remained transfusion-dependent but clinically stable, with normal organ function. At five years, he developed headaches and a single seizure episode; contrast-enhanced CT of the head demonstrated a large left temporal ex vacuo cyst with no evidence of acute hemorrhage, consistent with sequelae of prior intracranial bleeding. He was commenced on antiepileptic therapy with good control, and underwent a transplant procedure once neurological stability was confirmed. In view of the chance of rebleeding into this space, a high threshold for platelet transfusion was kept.

The child underwent haploidentical HSCT using his father (5/10 HLA match) as the donor. Conditioning included rituximab 375 mg/m² (day -8), rATG 4.5 mg/kg total (days -6 and -5), fludarabine 40 mg/m²/day (days -4 to -1), cyclophosphamide 14.5 mg/kg/day (days -4 and -3), and TBI 4 Gy (day -1). The PBSC graft contained CD34⁺ cells 18×10⁶/kg and CD3⁺ cells 2×10⁸/kg. GVHD prophylaxis comprised PTCy (50 mg/kg/day on days +3 and +4), cyclosporine (target trough 150-200 ng/mL), and MMF until day +28, followed by tapering.

The early post-transplant period was marked by transient febrile neutropenia and mild hemorrhagic cystitis, both resolving with conservative management. Neutrophil engraftment occurred on day +14, and the final platelet transfusion was on day +11. On day +22, he developed grade II acute gut GVHD, confirmed histologically and successfully treated with oral prednisolone (2 mg/kg/day) and budesonide (3 mg twice daily). He was discharged on day +34 with full donor chimerism and normal counts. At last follow-up (day +238; 15 March 2026), he remains clinically stable and transfusion independent.

Summary of transplant course

Transfusion Strategy

**Figure 3:**
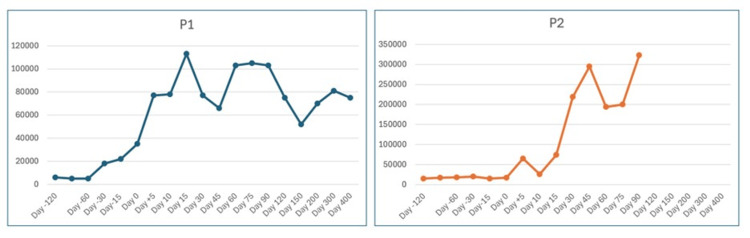
Salient clinical, genetic, and transplant characteristics of patients with congenital amegakaryocytic thrombocytopenia

Given both children's history of prior intracranial hemorrhage, an intensified platelet support protocol was implemented. Single-donor apheresis platelet transfusions were administered to maintain platelet counts above 40-50 × 10⁹/L throughout conditioning and the cytopenic phase until engraftment. Transfusions were typically given every 3-4 days or as needed based on daily count monitoring, as opposed to the standard practice of transfusing at a threshold of <20 × 10⁹/L. This strategy aimed to minimize re-bleeding risk and optimize hemostatic stability during conditioning-related endothelial injury and aplasia. It proved both feasible and safe, with no new hemorrhagic events observed. Serial platelet counts of both patients before and after haploidentical HSCT are illustrated in Figure 3.

P1 (left) and P2 (right) demonstrate trends in peripheral platelet counts from 120 days pre-transplant through 400 days post-HSCT. Both children showed profound thrombocytopenia prior to HSCT, with a rise in platelet counts with engraftment and sustained independence from transfusion. P1 achieved platelet recovery by day +14 and P2 by day +11 post-HSCT.

Infection Surveillance

Both patients underwent intensive viral monitoring post-HSCT. Quantitative RT-PCR assays for cytomegalovirus (CMV), Epstein-Barr virus (EBV), and adenovirus were performed weekly during the first three months and every two weeks thereafter until T-cell reconstitution was achieved. One patient experienced clinically significant viral reactivation, which was treated. None had graft failure, sinusoidal obstruction, worsening of liver function due to hepatitis C, or transplant-related mortality during the early follow-up period.

Neurological and Long-term Follow-up

Both children remain neurologically stable with no recurrence of seizures or focal deficits. Antiepileptic medications are being continued, with a plan to taper one year after the last seizure episode, following documentation of a normal EEG. Cyclosporine tapering is planned after one year post-HSCT, contingent on stable chimerism and absence of GVHD. This gradual immunosuppression withdrawal strategy, standard in non-malignant transplants, minimizes the risk of chronic GVHD while supporting immune recovery.

Immunomodulation

An important distinction between the two cases lies in the immunomodulatory strategies employed prior to transplantation. In the first child, bortezomib was administered pre-transplant to address platelet transfusion refractoriness secondary to alloimmunization. Given the child’s prolonged transfusion dependence, alloantibody formation against HLA and platelet antigens was suspected, leading to poor post-transfusion increments despite single-donor platelet support. Bortezomib, a proteasome inhibitor with plasma cell-depleting activity, was used in four weekly doses and resulted in improved transfusion responses prior to conditioning. This intervention helped achieve adequate platelet recovery and facilitated safe progression to HSCT.

In contrast, rituximab was incorporated into the conditioning regimen of the second patient as a preemptive measure to prevent post-transplant immune-mediated complications. As part of our institutional protocol for haploidentical HSCT in non-malignant disorders, rituximab (375 mg/m² on day -8) is routinely used to deplete B cells and reduce the risk of Epstein-Barr virus (EBV)-driven post-transplant lymphoproliferative disease (PTLD) and alloimmune cytopenias. Its inclusion in this case was particularly relevant given the child’s history of multiple transfusions and consanguinity, both of which increase the likelihood of alloimmune sensitization.

Rituximab was not included in the conditioning of the first child because, at the time of his transplant, the child had already received bortezomib for alloimmune platelet refractoriness, which effectively reduced circulating plasma cells and obviated the need for additional B-cell-directed therapy. Hence, rituximab was selectively withheld, and immune surveillance, including serial EBV PCR monitoring, was adopted instead.

Despite high-risk features, including prior intracranial bleeding, iron overload, and a haploidentical donor setting, both patients achieved sustained engraftment, transfusion independence, and stable donor chimerism without veno-occlusive disease or major infections. These results highlight that haploidentical HSCT with PTCy is a safe and effective curative approach for CAMT, even in children with prior neurological injury and advanced marrow aplasia, when managed with tailored supportive care and vigilant monitoring.

## Discussion

CAMT is a rare inherited bone marrow failure syndrome for which HSCT remains the only curative treatment. Transplant experience in CAMT has primarily been limited to matched sibling or unrelated donor settings, with outcomes improving significantly in recent years due to advances in conditioning, graft manipulation, and supportive care. The largest registry analysis of transplanted CAMT patients reported an overall survival of approximately 86% and graft-failure-free survival of 83%, with most recipients receiving bone marrow or cord blood grafts from HLA-matched donors. Historically, outcomes for mismatched transplants were less favorable, with high rates of graft failure and severe GVHD [11]. However, the introduction of post-transplant cyclophosphamide has dramatically improved haploidentical HSCT outcomes, as demonstrated in the present cases, where both children achieved sustained engraftment without transplant-related mortality [11].

Haploidentical transplantation has greatly expanded donor availability for children lacking HLA-matched donors, but published experience in CAMT remains extremely limited. Earlier attempts at haploidentical HSCT were associated with high rates of graft rejection and severe GVHD; however, the introduction of post-transplant cyclophosphamide (PTCy) has markedly improved outcomes, enabling safe and effective use of mismatched donors. In children with primary immunodeficiencies and other inherited disorders, haplo-HSCT with PTCy has demonstrated high rates of engraftment and encouraging long-term survival [16]. These developments have laid the foundation for extending this approach to non-malignant bone marrow failure syndromes such as CAMT [17-19].

The present report describes, to our knowledge, one of the earliest reports from India on haploidentical HSCT in genetically confirmed CAMT and among the very few globally in which both children had a history of intracranial hemorrhage prior to transplantation.

Intracranial hemorrhage in CAMT is associated with increased transfusion burden, predisposition to platelet alloimmunization and refractoriness, and may heighten the risk of complications during the cytopenic phase of HSCT [5]. However, published evidence supporting this exclusion is lacking. Both patients in our report tolerated myeloablative conditioning and transplantation without neurological deterioration, illustrating that prior intracranial bleeding should not preclude curative therapy when lesions are stable and peri-transplant neuroprotection is meticulous.

Key aspects of the cases

Feasibility of Haploidentical HSCT With PTCy

Both children achieved neutrophil engraftment by day +14 and platelet independence within two weeks using paternal haploidentical peripheral blood stem cell grafts. The absence of graft failure or transplant-related mortality demonstrates that modern haploidentical HSCT can achieve outcomes comparable to those of matched donor transplants in CAMT.

Advanced Marrow Failure at Transplantation

The first child had progressed to complete aplasia with a heavy transfusion burden and iron overload, yet engrafted successfully and remains transfusion independent. This underscores that haploidentical HSCT remains viable even in advanced disease when conditioning and supportive measures are carefully optimized. The case also highlights the importance of molecular testing in all children with aplastic anemia to identify underlying inherited marrow failure syndromes such as CAMT.

Low Early Toxicity and Manageable GVHD

Neither child developed veno-occlusive disease, thrombotic microangiopathy, or severe infections. Both experienced grade II acute GVHD, which resolved with corticosteroids and continuation of cyclosporine. Intensive viral surveillance with weekly adenovirus, CMV, and EBV PCR during the first three months, followed by fortnightly monitoring until immune reconstitution, likely contributed to early detection and prevention of viral complications.

Optimized Peri-Transplant Supportive Care

Both patients received an intensified platelet transfusion strategy due to their prior intracranial bleeding. Single-donor apheresis platelet transfusions were given once or twice weekly during conditioning and the cytopenic phase to maintain platelet counts above 40-50 × 10⁹/L. This approach prevented further bleeding and ensured hemostatic stability. Rituximab was included in the conditioning of the second patient as part of institutional practice for high-risk haploidentical HSCT to mitigate the risk of EBV-related complications.

Post-transplant Neurological and Immune Monitoring

Both children remain neurologically intact and continue on maintenance antiepileptics, with tapering planned one year after the last seizure, following a normal EEG. Cyclosporine tapering is also planned at one-year post-HSCT, consistent with standard protocols for non-malignant diseases, provided donor chimerism remains stable, and GVHD is absent. This gradual tapering minimizes chronic GVHD risk while supporting immune reconstitution.

Donor Access and Equity in Resource-Limited Settings

In countries with limited access to matched donors, haploidentical HSCT with PTCy provides a cost-effective, logistically feasible, and curative option for children with CAMT when performed in experienced centers with comprehensive supportive care.

Collectively, these findings reinforce that haploidentical HSCT with PTCy can be safely and effectively performed in CAMT, even in the presence of high-risk features such as prior intracranial hemorrhage or advanced marrow aplasia. The outcomes observed in this report are consistent with broader pediatric haploidentical HSCT data, which show comparable survival to matched donor transplantation and acceptable toxicity profiles.

Given the rarity of CAMT and other inherited marrow failure syndromes, the establishment of multicenter registries is critical to track longitudinal outcomes, optimize transplant timing, and identify regional challenges. Such registries are especially important in low- and middle-income countries, where delayed diagnosis, limited molecular testing, and donor scarcity remain major barriers. Systematic registry-based follow-up would allow benchmarking of outcomes and help refine best practices across centers [20].

Limitations

The main limitations of this report are its small sample size (n=2), single-center experience, and variable follow-up duration (555 days for Case 1, 238 days for Case 2). Longer follow-up is needed to assess late effects, including chronic GVHD, growth and development, and gonadal function after TBI-containing conditioning. Nevertheless, detailed reports such as these are essential to guide clinical decision-making and hypothesis generation in ultra-rare pediatric disorders.

Implications for practice and research

Clinicians should maintain a high index of suspicion for inherited marrow failure syndromes in infants with severe thrombocytopenia and pursue early molecular confirmation. Timely genetic diagnosis facilitates earlier HSCT referral before the onset of marrow aplasia or organ damage. Where matched donors are unavailable, haploidentical HSCT with PTCy offers a feasible and effective curative strategy when coupled with multidisciplinary transplant care and intensive supportive management. Future research should focus on the creation of international CAMT registries, comparative studies of conditioning regimens and graft sources, and long-term evaluation of neurodevelopmental outcomes following HSCT in children with prior intracranial bleeding.

## Conclusions

Haploidentical peripheral blood stem cell transplantation using PTCy-based GVHD prophylaxis can achieve rapid and durable hematopoietic recovery in children with CAMT, including those with prior intracranial hemorrhage and advanced marrow failure. However, successful outcomes depend on experienced multidisciplinary care, meticulous peri-transplant support (including intensified platelet transfusion), and careful patient selection. Although larger multicenter datasets are needed to refine best practices, this experience supports that prior neurological injury should not preclude curative transplantation in CAMT. In this rare disease, early recognition and carefully navigated haploidentical HSCT can be lifesaving.
